# Improving delivery of secondary prophylaxis for rheumatic heart disease in remote Indigenous communities: study protocol for a stepped-wedge randomised trial

**DOI:** 10.1186/s13063-016-1166-y

**Published:** 2016-01-27

**Authors:** Anna P. Ralph, Clancy Read, Vanessa Johnston, Jessica L. de Dassel, Kerstin Bycroft, Alice Mitchell, Ross S. Bailie, Graeme P. Maguire, Keith Edwards, Bart J. Currie, Adrienne Kirby, Jonathan R. Carapetis

**Affiliations:** Menzies School of Health Research, Darwin, NT Australia; Telethon Kids Institute, University of Western Australia, and Princess Margaret Hospital for Children, Perth, WA Australia; Charles Darwin University, Darwin, NT Australia; Baker IDI Heart and Diabetes Institute, Melbourne, VIC Australia; Paediatric Department, Royal Darwin Hospital, Darwin, NT Australia; National Health and Medical Research Council Clinical Trials Centre, University of Sydney, Sydney, NSW Australia

**Keywords:** Acute rheumatic fever, Rheumatic heart disease, Continuous quality improvement, Health-systems research, Stepped-wedge design

## Abstract

**Background:**

Rheumatic heart disease (RHD), caused by acute rheumatic fever (ARF), is a major health problem in Australian Aboriginal communities. Progress in controlling RHD requires improvements in the delivery of secondary prophylaxis, which comprises regular, long-term injections of penicillin for people with ARF/RHD.

**Methods/Design:**

This trial aims to improve uptake of secondary prophylaxis among Aboriginal people with ARF/RHD to reduce progression or worsening of RHD. This is a stepped-wedge, randomised trial in consenting communities in Australia’s Northern Territory. Pairs of randomly-chosen clinics from among those consenting enter the study at 3-monthly steps. The intervention to which clinics are randomised comprises a multi-faceted systems-based package, in which clinics are supported to develop and implement strategies to improve penicillin delivery, aligned with elements of the Chronic Care Model. Continuous quality improvement processes will be used, including 3-monthly feedback to clinic staff of adherence rates of their ARF/RHD clients.

The primary outcome is the proportion of people with ARF/RHD receiving ≥80 % of scheduled penicillin injections over a minimum 12-month period. The sample size of 300 ARF/RHD clients across five community clusters will power the study to detect a 20 % increase in the proportion of individuals achieving this target, from a worrying low baseline of 20 %, to 40 %. Secondary outcomes pertaining to other measures of adherence will be assessed. Within the randomised trial design, a mixed-methods evaluation will be embedded to evaluate the efficiency, effectiveness, impact and relevance, sustainability, process and fidelity, and performance of the intervention. The evaluation will establish any causal link between outcomes and the intervention. The planned study duration is from 2013 to 2016.

**Discussion:**

Continuous quality improvement has a strong track record in Australia’s Northern Territory, and its use has resulted in modest benefits in a pilot, non-randomised ARF/RHD study. If successful, this new intervention using the Chronic Care Model as a scaffold and evaluated using a well-developed theory-based framework, will provide a practical and transferable approach to ARF/RHD control.

**Trial registration:**

Australian New Zealand Clinical Trials Registry: ACTRN12613000223730. Date registered: 25 February 2013

**Electronic supplementary material:**

The online version of this article (doi:10.1186/s13063-016-1166-y) contains supplementary material, which is available to authorized users.

## Background

Acute rheumatic fever (ARF) and its sequel, rheumatic heart disease (RHD), remain important causes of morbidity and mortality in areas of socioeconomic deprivation. Rates in Australia’s remote-dwelling Aboriginal populations are amongst the highest reported globally; the rate in school-aged Aboriginal children in Australia’s Northern Territory (NT) in 2005–2009 was estimated at 150–380 per 100,000 [1–3]. Numerous steps have been taken to attempt to reduce the burden of ARF/RHD in Australia. In the NT, this has included: making ARF a notifiable disease; developing and funding a register and control programme to coordinate patient care; developing freely available patient resources, staff training modules and management guidelines [2]; developing a new smartphone application for ARF diagnosis to help health care staff unfamiliar with the disease [4]; and other initiatives. However, the barriers to ARF/RHD prevention remain prevalent and are multi-factorial.

ARF is a preventable disease caused by an abnormal host immune response that can develop after infection with group A streptococcus, a common cause of pharyngitis and skin sores. Children aged 5–14 years are most at risk. In remote NT Aboriginal communities, exposure to pathogenic group A streptococcal strains is frequent, but only a small proportion of individuals develop the autoimmune response causing ARF. Recurrent ARF episodes cause cumulative heart valve damage, leading to RHD and its serious complications: heart failure, atrial fibrillation, requirement for major cardiac surgery for valvular repair, anticoagulation, thrombotic and haemorrhagic complications including stroke, endocarditis and premature mortality. A recent NT study demonstrated that after a first ARF diagnosis, 61 % of people developed RHD within 10 years, and of those nearly 30 % progress to heart failure within 5 years [5]. Given the young age of disease onset the morbidity burden and cost to society is very high.

### Secondary prophylaxis

Strategies for ARF prevention exist at the primordial, primary, secondary and tertiary levels [3]. Secondary prevention is the strategy with the best-proven efficacy and cost-effectiveness [6, 7]. This comprises regular antibiotic prophylaxis to prevent further streptococcal infections, and hence ARF recurrences, among individuals after their first ARF diagnosis. The standard recommended antibiotic regimen is 4-weekly intramuscular injections of benzathine (long-acting) penicillin G, for 10 years after the last ARF episode or until age 21, whichever is longer. Longer durations are required in people with moderate to severe RHD. This strategy significantly reduces ARF recurrence rates compared with placebo [8] or oral penicillin [7]. It forms the cornerstone of the World Health Organisation’s RHD prevention strategy globally [9], and the Australian guidelines locally [2].

It is believed that the most effective way to govern delivery of secondary prophylaxis, as well as other elements of ARF/RHD clinical care, is via register-based control programmes [10]. The NT RHD Control Programme and Register were established in the north of the NT in 1997.

A key challenge exists in improving adherence to secondary prophylaxis. Individuals should ideally receive every scheduled injections to ensure continual protection from streptococcal infection and subsequent ARF episodes. A more achievable target is ≥80 % of scheduled injections [2], yet the proportion of clients with ARF/RHD achieving this target in the NT in 2009 was only 23 % [11].

### Adherence

Adherence is a major theme in the discussion of chronic disease management in Australian Indigenous people [12]. Determinants of adherence can be understood within the key domains of socioeconomic factors, health systems, medications including adverse effects, clients and the medical condition itself [13]. Adherence to ARF/RHD secondary prophylaxis, a directly-observed, health care provider-administered injection, includes unique additional challenges: the treatment is an often painful needle-prick; health care staff turnover in remote clinics is exceptionally high, and their knowledge of ARF/RHD is often poor; patient populations are young and mobile; health literacy tends to be low; and furthermore, patient traditional knowledge, world view and expectations of health care differ from what is offered by traditional Western medical models [14–16]. Two small studies in northern Australia found that adherence among Aboriginal clients with ARF/RHD appeared unrelated to their health literacy or to fear of injections, but instead to their relationship with primary care staff and the presence of active recall systems [14, 15].

At the health centre level, important determinants of adherence identified in other studies include a culture free from institutional racism, with a degree of community control, strong leadership and opportunities for building the capacity of local workforce [17, 18]. A Cochrane review of the international literature on adherence support initiatives found only modest benefits, even from quite labour-intensive strategies [19]. Such findings set a challenge for studies such as ours. However, adherence to an administered injection provides an opportunity for creative, multi-dimensional health-centre-based strategies, and the existing evidence base for improving health service delivery in our setting (see below) provides optimism that a carefully planned strategy can be successful.

### The chronic care model

The logical approach to improving secondary prophylaxis uptake in the NT setting would comprise an intervention targeting the recognised challenges to secondary prophylaxis adherence, with a key focus on health service delivery. We have selected the Chronic Care Model (CCM) as a scaffold for implementing an intervention package at participating health centres. The CCM is a comprehensive system approach for chronic disease management, incorporating six domains: health systems, delivery system design, decision support, clinical information systems, self-management support and community linkages [20]. The CCM is appropriate since although individual ARF episodes are by definition acute, the required management (long-term regular secondary prophylaxis and engagement with health services) is synonymous with chronic disease management, and the long-term complication, RHD, is chronic. The advantage of the CCM is that it is a ‘whole-system’ model targeting both the client and the health service. It is also the current framework for the NT Chronic Disease Strategy and, therefore, situates the study well within current practices; this should enhance local transferability if the trial is successful [21].

### Continuous quality improvement processes

One approach which has been successfully adopted in the NT to achieve systems change at health clinics has been systematic continuous quality improvement (CQI) activities [22]. CQI strategies engaging primary health staff have been shown to improve health outcomes in remote clinics in the NT [23]. Health systems improvements providing a ‘comprehensive-care programme’ were shown to reduce ARF rates in the USA as long as four decades ago [24], and reductions in ARF burdens have been achieved in Cuba and the Caribbean with programmes combining community education strategies with health care improvements (e.g. development of a register, education for health staff) [25, 26].

Specifically, regarding ARF/RHD in the NT, we have shown that application of a CQI process over 3 years at six remote clinics in the NT led to small but statistically significant improvements in some proximal outcome measures for ARF/RHD patients [11]. Tools used included an audit tool allowing clinics to monitor their delivery of services to ARF/RHD clients, and a ‘systems assessment tool’ addressing systemic impediments to RHD service delivery. These tools are contained within the National Centre for Quality Improvement in Indigenous Primary Health Care (‘One21seventy’) package [27]. Our study showed substantial variability between sites in their delivery of RHD services, and in the strategies they used to promote RHD service delivery. Factors associated with performance in these clinics are listed in Table 1 (listed in order of amenability to change). Many could potentially be addressed using existing resources.

**Table 1:** Summary of factors influencing performance of six remote Northern Territory health centres in delivering services to clients with acute rheumatic fever/rheumatic heart disease (ARF/RHD)

Determinants of relatively good performance	Determinants of relatively poor performance
• Clear allocation of responsibility for rheumatic heart disease (RHD) care among health centre staff • Good regional management – commitment to continuous quality improvement (CQI), resourcing for CQI • Effective feedback and management action in response to feedback from CQI process • Good Aboriginal health worker involvement in health centre operations • Good outreach arrangements – including drivers, Aboriginal health workers • Public health-oriented chronic disease support from regional level to health centres • Staff stability and continuity, including availability of experienced general practitioner	• Client flows in health centres do not direct ARF/RHD clients to staff responsible for RHD care • Lack of clear allocation of responsibility for RHD care • Lack of effective outreach services • Changes and inefficiencies in clinical information systems • Lack of regular/stable staffing, including general practitioner service • Health centre management turnover, unstable management structure • Larger number of clients, complexities of urban environment

Another recent study in NT remote clinics implemented a Chronic Conditions Management Model, comprising decision support for health care staff and provision of simple, coloured (‘traffic light’) reports to monitor the alignment of a clinic’s performance with goals. This was associated with improved patient outcomes [28]. The authors concluded that even in Australia’s most challenging primary health care contexts, improvements were achievable by identifying clear goals, and providing staff with support and feedback including reports generated from the clinic’s own data [28]. CQI processes, under the CCM domain of clinical information systems, will be integrated into our study’s intervention package; this will include 3-monthly feedback to clinic staff of adherence rates of their ARF/RHD clients.

### Objectives

Given the major burden posed by ARF/RHD for Aboriginal people in the NT, the proven benefit of secondary prevention with regular penicillin injections, but the challenges of delivering secondary prevention effectively, we therefore aim to improve the uptake of secondary prophylaxis among people with ARF/RHD. The predicted consequences of improved uptake are reduced rates of recurrent ARF episodes and mitigation of RHD development or progression. The primary objective is to test whether an intervention to optimise health systems can improve adherence to secondary prophylaxis for RHD. Objectives of the mixed-methods evaluation are to test efficiency, effectiveness, impact and relevance, sustainability, process and fidelity, and performance of the intervention.

## Methods/Design

### Study design

**Fig. 1:**
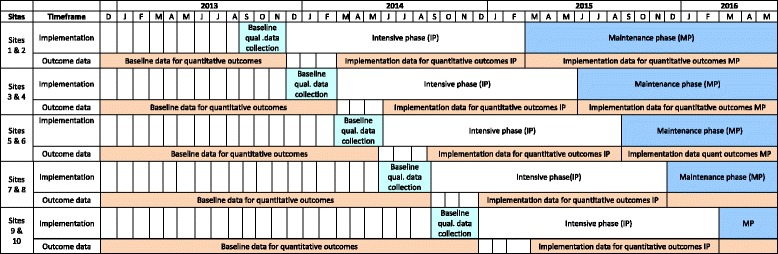
Study design demonstrating the stepped-wedge process. The stepped-wedge design involves the sequential roll-out of an intervention over periods of time; by study completion, all participating health centres will have received the intervention, for a 15-month period

This is a stepped-wedge, community randomised trial with an open cohort design. Key characteristics of the trial are shown in Table 2 and Fig. 1. A stepped-wedge trial is a type of cluster randomised trial in which ‘clusters receive the intervention at different time points, the order in which they receive it is randomised, and data are collected from clusters over time’ [29]. The open cohort means that individuals may leave during the trial (no longer require ARF/RHD secondary prophylaxis, die or move to a non-study community), or may become eligible during the trial (receive a new diagnosis of ARF/RHD requiring secondary prophylaxis). Steps of 3 months’ duration were chosen to provide a feasible total study duration whilst providing adequate time for project staff to accomplish intensive visits for incoming clusters. A lag time of 3 months has been anticipated between commencing the intervention and onset of a perceivable effect, hence data collection for quantitative measures during the intervention period will commence 3 months after the onset of the intensive phase for each cluster, providing a 12-month period. Since the intervention is applied at the health centre level, within-cluster correlation has been accounted for in the sample size calculation. Observations will be made on individuals (number of penicillin injections, as documented routinely by the RHD Register, before, during and after the intervention), so the effect of the intervention will be assessed on a within-client basis. Within the randomised trial design, mixed-methods evaluation will take place to address the study objectives.

**Table 2:** Characteristics of the ‘Improving Secondary Prophylaxis’ stepped-wedge trial

Term/Trial characteristic	Definition
Unit	Aboriginal health centres
Cluster	Pairs of health centres
Individuals	People with ARF/RHD who require secondary prophylaxis with penicillin, whose primary health centre is enrolled in the study
Timing of start of exposure	Groups of individuals (all individuals managed by a given community clinic) are first exposed at one of a number of discrete time points
Duration of exposure	Fixed length: 15 months per clinic
Measurement	Repeated measurements from individuals: record of every penicillin injection received, as documented in the ARF/RHD Register
Duration of trial	3.5 years
Number of clinics per cluster	2
Total number of clusters	5
Pre-roll out period	11 to 25 months
Roll out period	15 months
Post-rollout period	3 to 15 months

*ARF* acute rheumatic fever, *RHD* rheumatic heart disease

### Study setting

The study is set in Australia’s NT (population: 243,700; population density 0.17/km^2^), with a high proportion of remote-dwelling Aboriginal people, for whom English is often a second language. Each community of adequate size has one health centre (clinic), the sole provider of health care for the community. Towns each have one Aboriginal Health Service but additional mainstream medical services are also available. There are over 80 individual Aboriginal communities with health centres in the NT; health centres are either community-controlled or operated by the local Government Department of Health. The NT is divided into two jurisdictions − Top End (which includes the regional capital) and Centre, with separate clinic-level governance and separate RHD Control Programmes.

### Inclusion criteria

Communities are eligible to participate if they have at least 10 individuals with ARF/RHD requiring secondary prophylaxis, are willing to participate, and provide written informed consent. We will seek sites representative of the NT as a whole including Top End and Central sites, community-controlled and government run, small and large, remote and regional.

Individuals from within consenting sites (health centre staff and ARF/RHD clients), and additional stakeholders or relevant experts, are enrolled for semi-structured interviews, to address the qualitative study objectives. Individuals are eligible if they meet criteria for the required interviewee type, and provide written informed consent.

### Consent process

Consent from health centres to participate in the study requires a signed partnership agreement between the health centre manager (government-run centres) or management board (community-controlled centres), and a study investigator. Discussions leading up to the signing of the agreement comprise various stakeholder meetings (e.g. with the organisation of Remote Medical Practitioners and with the body overseeing community controlled centres), plus teleconferences and visits to clinics by research staff, and provision of detailed materials to health centre staff and relevant community organisations, to assist them in deciding on participation or not. Consenting health centres are able to withdraw consent at any stage without prejudice. Consent from individuals for their anonymised RHD Register data to be used to calculate primary and secondary outcome measures is not sought. The ethics committee agreed that over-arching consent at clinic/community level is appropriate.

Consent from individuals to participate in interviews is sought by project officers using written and verbal materials, with verbal information in an Australian Indigenous language where appropriate. For individuals aged under 15 years, consent is sought from a parent/guardian, and verbal assent from the interviewee. The ethics committee agreed that for individuals aged 15 years or older, participation in an interview about their medical condition and treatment, without parental consent, was appropriate. All written documentation is de-identified.

### Implementation and project phases

The intervention comprises a set of activities developed and implemented by health centres, aligned under the six themes of the CCM. An additional file shows in detail the types of activities which health centres may adopt (see Additional file 1: Table S1). Implementation is supported by research staff, and progress is monitored using CQI processes. This includes 3-monthly adherence rate reports, presented using simple, coloured graphics and written summaries. Action plans are amended where necessary in response to these reports.

The first (now completed) phase of the project comprised community engagement for recruitment of consenting health centres. The main intervention phases then comprise: (1) *Baseline* data collection and planning (3 months); (2) *Intensive* implementation phase (15 months); (3) *Maintenance* implementation phase (up to 15 months depending on site’s start date) (Fig. 1). In accordance with stepped-wedge trial design, the intervention is implemented at the level of the community/health centre, but measured by its impact on individuals, i.e. clients with ARF/RHD (primary endpoint) and health centre staff (secondary endpoints) [29].

During the 3-month *baseline phase*, project team members visit the health centre for two approximately 5-day visits, one for data collection; the second for planning. Qualitative data including barriers to delivery of secondary prophylaxis at the health centre are identified and recorded through workshops facilitated by the project officer, and an action plan is developed. All site visits, meetings and other arrangements are planned in collaboration with the community stakeholders and health centre staff to mimimise the impact on routine service delivery.

At commencement of the 15-month *intensive phase*, items in the action plan start being implemented, according to clinic priorities. Potential activities for inclusion in the action plan are listed in Table 3. While necessarily being tailored to local situations, we aim for homogeneity of approaches to ensure similarity of the intervention across sites; this is aided by aligning activities under the six themes of the CCM. All efforts are made to ensure that the exposure to (‘dose’ of) the intervention is uniform across clusters: the duration of intensive phase is identical for each cluster, and the number of scheduled site visits or of episodes of telephone support provided by project staff is uniform. In supporting implementation of the intervention, the research team work closely with health centre staff, CQI facilitators and the NT RHD Control Programme. The action plan is implemented by the health centre staff, supported by the project team. A project team member visits the community monthly, for 1 to 3 days per trip. Visits are supplemented with email and telephone contact.

**Table 3:** Potential activities for inclusion in health centre’s action items, grouped under the six streams of the Chronic Care Model

Health system	Delivery system design	Decision support	Clinical information systems	Self-management	Community linkages
Establish a multi-disciplinary RHD working group in health centres comprised of health centre staff and key stakeholders	Allocate, confirm and document responsibility for ARF/RHD care among health centre staff to facilitate planned care interactions and follow-up	Integrate evidence-based guidelines and decision support aids for ARF/RHD into daily clinical practice	Monitor performance of practice team and care system in relation to ARF/RHD care using CQI processes	Up-skill health centre staff in self-management support techniques through engagement with NT Department of Health training activities	Partner with community resources to support timely delivery of SP to ARF/RHD clients
Support the NT Department of Health Steering Committee within NT Department of Health to coordinate RHD care	Streamline care for ARF/RHD clients through: o Fast-tracking at reception o Process of client identification for opportunistic delivery of SP o Prioritisation of case management for ARF/RHD clients with poor adherence	Ensure health centre staff are trained regularly on ARF/RHD care with an emphasis on SP planning and delivery	Establish and refine systems to monitor and report ARF/RHD client data regularly to health centre staff to facilitate care planning	Establish/strengthen group self-management support programme for ARF/RHD clients, facilitated by health centre staff where expertise available	Strengthen health literacy activities in communities around RHD
			Review and strengthen active systems of reminders and recalls for SP for health centre staff and ARF/RHD clients	Explore sustainable incentives to ARF/RHD clients for adhering to SP	Develop better understanding of community knowledge of and attitudes towards ARF/RHD care

*ARF* acute rheumatic fever, *CQI* continuous quality improvement, *NT* Northern Territories, *RHD* rheumatic heart disease, *SP* secondary prophylaxis

During the *maintenance phase*, following 15 months of intensive implementation, the intention is for the clinic’s agreed action plan to be sustained. One project team member visits quarterly and email/telephone contact is made monthly.

A quarterly study newsletter (electronic and paper format) is circulated to participating sites, investigators and other interested parties, to promote enthusiasm for the project, provide cross-fertilisation of ideas between participating sites to include in activity plans, congratulate sites on their progress and share news of any relevant developments in ARF/RHD management.

This project is specifically designed with sustainability and transferability in mind, deliverable within existing health care structures and resources.

### Outcome measures

The primary outcome measure is proportion of clients receiving ≥80 % of their scheduled benzathine penicillin G injections over the 12-month intervention period, compared with the pre-intervention period. This will be calculated using routinely-collected RHD Register data. Data will be anonymised prior to analysis. All outcome measures are shown in Table 4.

**Table 4:** Trial outcome measures

Primary outcome	• Proportion of clients receiving 80 % or more of their scheduled benzathine penicillin G (BPG) injections over the 12-month intervention period, compared with the pre-intervention period
Secondary outcomes	• The proportion of scheduled injections that a client receives in a 12-month period • The average number of days at risk. ‘Days at risk’ is the number of days over 28 days between scheduled injections in a month. For clients on 3-weekly injections, ‘Days at risk’ is the number of days over 21 days between scheduled injections • Proportion of clients receiving at least 90 % of scheduled BPG injections over a minimum 12-month period • Proportion of clients receiving 50–79 % and <50 % of scheduled BPG injections over a minimum 12 month period • Recurrence rate and proportion of ARF episodes that are recurrences, compared to non-participating communities and to the whole jurisdiction • Impact of the intervention on RHD clients’ experience of care including their perception and understanding of the disease and its management • Improvement in delivery of other services for RHD clients • Effect of the programme on delivery of other routine services

*ARF* acute rheumatic fever, *RHD* rheumatic heart disease

### Sample size

Using a stepped-wedge design with a 3-month period for each step, with enough communities to provide at least 30 subjects at each step, and taking into account within-cluster correlation, we calculated that 300 clients will provide 90 % power and a two-sided significance level of 5 % to detect an increase from 20 % of subjects receiving ≥80 % of their scheduled penicillin injections to 40 %. This number of clients will provide more than 90 % power to detect a doubling of the rate, if the pre-intervention rate is higher than 20 %. We estimated that health centres at up to 12 communities would be required to ensure a minimum of 300 clients diagnosed with ARF/RHD requiring secondary prophylaxis with penicillin.

The sample size is conservative (i.e. potentially larger than required) as it was calculated based on estimated between-client differences rather than within-client differences, which are usually smaller. That is, since change within a person is what is being measured, and within-client differences (variability within a person) are usually less than between-client differences (variability between people), and lower variance is associated with a lower required sample size, this study will, therefore, be well-powered. We have chosen an ambitious target (doubling the proportion of clients receiving 80 % or more of their scheduled injections) because a complex intervention such as this needs to show a substantial, clinically meaningful benefit if it is to be more widely recommended. The ratio of female to male ARF/RHD clients is approximately 1.3 to 1. Randomisation will be by community, so all ARF/RHD clients requiring secondary prophylaxis in each community will automatically be included.

### Randomisation

Clusters comprise pairs of health centres, entering the study at 3-monthly steps in random order. The random allocation code was generated by the statistician investigator (AK), using StataCorp 2015 Stata Statistical Software: Release 14 (StataCorp LP, College Station, TX, USA). The pairing of health centres was chosen prior to randomisation on grounds of geographical proximity to facilitate travel and minimise project costs, given the very large distances involved. The investigators and the health centres will be informed of the next two centres to commence after the previous centres have been enrolled, to maximise allocation concealment, but also permit adequate time for logistical planning.

This design ensures that all health centres will receive the potential benefits of the intervention while using a robust design (randomisation to intervention timing), and maximising efficiency (study personnel can provide intensive support for different health centres sequentially) [30].

### Data

Data sources chiefly include the NT RHD Register for quantitative analyses, and semi-structured interviews and project officer reports for qualitative analyses. Both provider and client perspectives will be sought, although as the intervention chiefly seeks to change health systems, there will be a particular focus on health provider perspectives. These plus the range of other data sources are listed in Table 5. Important variables included in the RHD Register which will be extracted include client sex, age, date and place of receipt of each penicillin injection during the study period and occurrence of any ARF episodes during the study period.

**Table 5:** Sources of data for addressing research objectives

Research objective	Data collection tools	Frequency
1. To test whether a model of care designed to optimise health systems improves adherence to secondary prophylaxis for RHD	• RHD Register data • Spreadsheet of time on intervention for each community	• Continuous
2. To assess the extent to which health clinics change their delivery of RHD care to align with the systems-based model and the barriers and enablers of organisational change	• Systems Assessment Tool (SAT), a component of the One21seventy tool RHD Continuous Quality Improvement package • Project Officer Reports – structured reports from project staff detailing implementation	• Baseline and post intensive phases • Completed at every site visit
	• Semi-structured interviews with clients/carers of clients, clinic managers, RHD coordinators, RHD programme staff, other relevant staff (chronic disease coordinators, NT Health Development public health nurses), using an interview guide for each group of participants	• Mostly baseline and post intensive phases
	• Project Officer Reports	• Completed at every site visit
	• Document review (e.g. meeting minutes, feedback reports from CQI audits)	• As arise
3. To explore the degree to which adopting the systems-based model improves processes of RHD care and adherence to secondary prophylaxis and which elements of the model are most effective in activating change	• RHD SAT and RHD Register	• Baseline and post intensive phases
	• Semi-structured interviews as described above	• Clients/carers: baseline and post intensive phases • Clinic staff and Control Programme staff: baseline and post intensive phases • Other relevant stakeholders: baseline and post intensive phases
	• Project Officer Reports	• Completed at every site visit
4. To explore environmental, organisational and team factors associated with success in achieving organisational and patient-level improvements in secondary prophylaxis for RHD	• RHD SAT and RHD Register	• Baseline and post intensive phases
	• Semi-structured interviews as described above	• Mostly baseline and post intensive phases
	• Project Officer Report	• Completed at every site visit
	• Document review (e.g. meeting minutes, feedback reports from CQI audits)	• As arise
5. To assess the impact of the systems-based model on other services for RHD clients	• RHD CQI audit of ARF/RHD clinical measures, a component of the One21seventy tool RHD Continuous Quality Improvement package	• Baseline and post intensive phases
6. To assess the impact of the systems-based model on other routine services delivered in the clinics	• NT Aboriginal health key performance indicators data	• Baseline and post intensive phases
7. To assess the impact of the intervention on clients’ experience of health care in relation to their ARF/RHD	• Interview guide for RHD clients	• Baseline and post intensive phases

*ARF* acute rheumatic fever, *CQI* continuous quality improvement *NT* Northern territories, *RHD* rheumatic heart disease

### Statistical methods plan for measuring outcomes

Most analyses will be undertaken using generalised linear mixed models. As the outcomes for clients within a community are not independent of each other, the method of analysis will take this correlation into account. The primary outcome is binary so will be analysed using this approach with a logit link. This analysis will allow a comparison of the effect in each community. Outcomes that are normally distributed will be analysed using the mixed-model approach to account for the within-community correlations.

For outcomes that can be measured accurately in shorter (1–3 month) time periods, trends over time can be assessed within these mixed-model approaches.

Any outcomes that are measured at a community level will be analysed using either a McNemar’s test for binary outcomes or a paired *t* test for normally distributed continuous outcomes. These analyses can be extended to incorporate the effect of covariates if necessary.

Secondary analyses will include models to assess client or community factors that are related to outcomes. Statistically significance will be understood to be attained when the *p* value is <0.05.

### Evaluation plan for measuring objectives

**Fig. 2:**
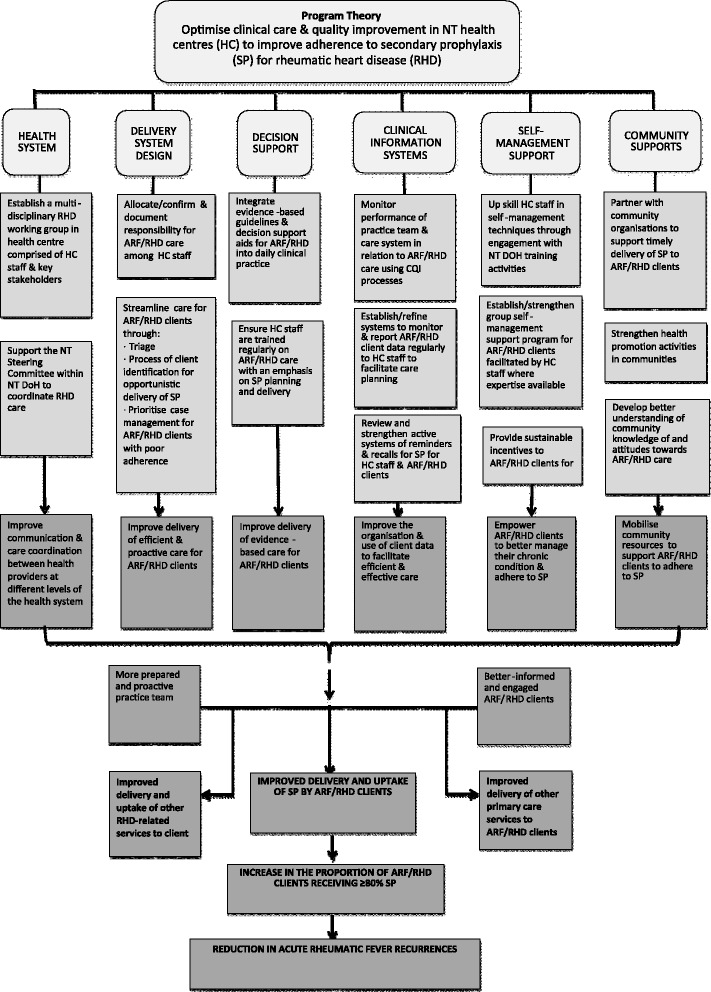
Programme theory. The programme theory (or project strategy) uses the six themes of the Chronic Care Model (health system, delivery system design, decision support, clinical information systems, self-management support and community supports) as the scaffold for activities to implement within the study intervention. A cascade of potential outcomes arising from these activities is shown, ultimately leading to increased adherence and thence, reduction in acute rheumatic fever recurrence rates

The evaluation framework is based on recommended strategies for evaluating complex interventions [31–33]. It seeks to answer the overall question of whether the intervention works, as well as the ‘hows’ and ‘whys’ of success or failure [31, 34]. The evaluation is based on a conceptual framework called programme theory [34–36]. The programme theory for this study (Fig. 2) is a systematic configuration of ‘prescriptive assumptions’ of the project (what action is required to improve adherence to ARF/RHD secondary prophylaxis?) and ‘descriptive assumptions’ (why will adherence be affected by these actions?). The CCM provides the scaffold to stipulate the cause-and-effect sequence through which actions affect outcomes [37].

**Fig. 3:**
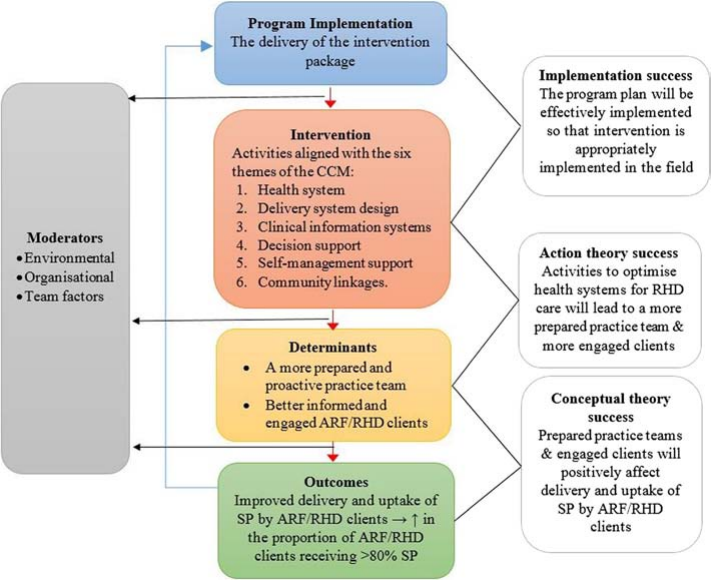
Evaluation framework. The central panel of this summarised schematic of the theory-driven evaluation framework illustrates the interplay between implementation (delivery of the intervention to clients, intervention (the agents of change that affect determinants), determinants (change mechanisms) and outcomes (improved adherence). Underpinning theories shown in the right hand panel are action theory (the intervention’s power to affect determinants), and conceptual theory (the determinant’s ability to affect outcomes). External moderators to be accounted for (the environment, organisation factors and team factors) are shown in the left hand panel

The successes of three domains will be tested (Fig. 3): implementation success (that the intervention was appropriately implemented); action theory success (that the intervention has successfully affected the causal variable in the conceptual theory); and conceptual theory success (that the intervention has successfully affected the causal variable *and* that the outcome variable has been successfully affected).

Evaluation measures will be based on research objectives (Table 5) under the categories of efficiency, effectiveness, impact, relevance and sustainability, as well as measures to examine implementation process, fidelity and performance. *Efficiency* measures will answer: ‘To what extent did health centres change their delivery of ARF/RHD care to align with the systems-based intervention?’ *Effectiveness* measures will answer: ‘To what degree did adopting the systems-based intervention improve processes of ARF/RHD care and adherence to secondary prophylaxis’ and ‘Which elements of the intervention were most effective in activating change?’ *Impact and relevance* measures will answer: ‘Did the intervention, a model of care designed to optimise health systems, improve overall adherence to secondary prophylaxis for ARF/RHD and minimise ‘days at risk’?’ *Sustainability* measures will answer: ‘Which of the activities and streams of the CCM were sustained during maintenance phase?’ *Process and fidelity* will be measured by asking: ‘What was the acceptability and completeness of implementation of the intervention package, and of individual items (measured from the health centre and client perspective)?’, ‘What were the barriers and enablers of implementation?’, ‘What were the barriers and enablers of organisational change?’ *Performance* will be measured by asking: ‘What were the factors associated with success in achieving organisational and client-level improvements in secondary prophylaxis for ARF/RHD?’ Answers to these questions will allow us to answer the question as to whether outcomes arise as a result of the intervention and what elements of the intervention contribute to its success or failure.

The analysis approach for qualitative data collected throughout the phases of the trial, including interviews and project officer reports, will be confirmatory (hypothesis-driven) [38]. An analytical framework will organise the data once it has been coded and analysed using thematic analysis. Data will be managed using Nvivo software (version 10, QSR International Pty. Ltd, Doncaster, VIC, Australia).

### Ethics

The study has received ethical approval from the Human Research Ethics Committee of the NT Department of Health and Menzies School of Health Research (approval number 2012–1756), and the Central Australian Human Research Ethics Committee (approval number 2013–126). Trial registration details are: Australian New Zealand Clinical Trials Registry: ACTRN12613000223730.

## Discussion

This will be the first randomised trial internationally to test a model of care to improve delivery of secondary prophylaxis for ARF and RHD. RHD is responsible for an unacceptably high burden of premature morbidity and mortality in Indigenous Australians, and disadvantaged populations globally [3]. Improved secondary prevention would make a major contribution to reducing this disease burden. This study is occurring within a coordinated body of work seeking to develop for the first time a comprehensive, evidence- based strategy for ending RHD [39]. Feasibility of the study is facilitated by the broad expertise of the investigator team, spanning clinical practice, quantitative research, statistical methods, qualitative methods, health services research and evaluation.

Community engagement and recruitment of consenting health centres took place during 2012 and 2013. The required sample size of ARF/RHD clients on secondary prophylaxis was exceeded after enrolment of 10 sites; in fact the number of total clients at study commencement within the first 10 sites to consent was 408 (minimum sample size = 300); hence, the study should be well-powered to determine any difference in the primary outcome measure. A broad representation of community sizes, locations and governance structures exists within the 10 participating communities.

Methodological challenges encountered during implementation of this study to date have arisen related to the stepped-wedge design, the type of ‘treatment’ to which clinics are randomised, and from the study environment. Time lags are an important challenge to address in stepped-wedge designs. We have observed that the time lag between intensive phase start date at a given site and when the intervention may plausibly start having measurable effects, not just on clinic processes but also on patients, is likely to be variable from site to site. We also recognise that clinics may struggle to develop and enact their action plan in accordance with study timelines and may, therefore, need to adjust subsequent study milestones at given sites accordingly.

A specific challenge and potential limitation for a trial in which the ‘treatment’ is an intervention package is that of fidelity − the degree to which the intervention is delivered as intended. Substantial efforts have been made to achieve homogeneity of the intervention across sites, including the components as devised and delivered at individual clinics, and the ‘dose’ (amount of support received by the project officers). However, there are inevitable variations, and we acknowledge that careful documentation of fidelity and dose are required to ensure that results will be interpretable regarding associations between intervention components and outcomes. However, such a challenge should not prevent complex interventions being subjected to rigorous study designs such as clinical trials.

Patient movements comprise another challenge. Aboriginal people frequently spend time in two or more communities. The RHD Register nominates one clinic as a person’s primary clinic (where the majority of their injections are administered) and secondary clinic(s) are also recorded. In documenting patient movements (new diagnoses, deaths, people moving from one site to another such that their primary clinic is reassigned), preliminary findings suggest a substantial number of movements are occurring. Such movements in and out of the cohort will need to be taken account of in analyses.

Characteristics of the study sites include very high staff turnover at health centres, and frequent disruption of clinic activities due to other priorities in communities, in particular funerals, which may result in closure of the clinic for some days in respect of ceremonial activities. We have found additional challenges in creating a metric for the measurement of staff turnover, and also in creating a metric for community remoteness or disadvantage. Health centre staff are busy with clinical commitments and complex acute care management, and there is the potential for them to suffer research fatigue due to the high number of studies being conducted at their sites. We are aware of this potential issue and have sought to demonstrate how this study can be directly beneficial to enrolled sites, and to be flexible in our approach; however, flexibility can pose a threat to intervention fidelity.

Improving the delivery of secondary prophylaxis is the major priority in ARF/RHD control in Australia and globally to reduce deaths and suffering from RHD. The model we are trialling is designed to fit within current structures of health service delivery, and to require minimal additional resources. The study design is rigorous, the primary endpoint (proportion who receive ≥80 % of injections) is of clinical importance for individual clients, and the evaluation will also allow us to determine the broader benefits of the intervention, along with any shortcomings. If successful, there is a high likelihood of the model being adapted into routine service delivery; the study investigators are well-aligned with policy-makers able to promote uptake of the research findings. Success in improving adherence in this study may also have important relevance to adherence to medications for other chronic diseases − leading causes of the large difference in life expectancy between Australia’s Indigenous and non-Indigenous populations. It will also provide guidance for improved RHD care internationally.

## Trial status

This trial is underway. Ongoing recruitment of participants for interviews and ongoing project officer site visits are in progress.

## Additional file

Additional file 1: Table S1. Suggested activities for each theme of the Chronic Care Model. This table provides a detailed description of the types of activities which participating health centres may adopt, in order to improve delivery of secondary prophylaxis for rheumatic fever/rheumatic heart disease. The suggested activities are categorised according to which theme of the chronic care model they best relate to. (DOCX 23 kb)

## Abbreviations

ARF: acute rheumatic fever
BPG: benzathine penicillin G
CQI: continuous quality improvement
DOH: Department of Health
NT: Northern Territory (Australia)
RHD: rheumatic heart disease
