# Supplementary material for: Improving delivery of secondary prophylaxis for rheumatic heart disease in remote Indigenous communities: study protocol for a stepped-wedge randomised trial
Source: Trials. 2016 Jan 27;17:51. doi: 10.1186/s13063-016-1166-y (PMC4729116; doi:10.1186/s13063-016-1166-y)
Supplement: Additional file 1: Table S1. — Suggested activities for each theme of the Chronic Care Model. This table provides a detailed description of the types of activities which participating health centres may adopt, in order to improve delivery of secondary prophylaxis for rheumatic fever/rheumatic heart disease. The suggested activities are categorised according to which theme of the chronic care model they best relate to. (DOCX 23 kb) [file 13063_2016_1166_MOESM1_ESM.docx]

**Supplementary Table: Suggested activities for each theme of the Chronic Care Model**

| **HEALTH SYSTEM**   - *Establish a multi-disciplinary* *Acute Rheumatic Fever (ARF) / Rheumatic Heart Disease (RHD) working group in the Health centre comprised of HC staff and key stakeholders*   *Who:* Northern Territory (NT) RHD Control Program staff, Health Centre (HC) manager, HC RHD Coordinator, Aboriginal Health Practitioner, a local significant community representative, Project Officer. Other staff as group see fit (e.g. drivers and reception staff, General Practitioner, specialists who regularly review the ARF/RHD clients, Health Development Unit staff, Area Service Manager, dentist).  *What:* 4-monthly community RHD working group meetings  *Why:* to collaboratively improve care coordination between health providers  *Data:* staff interviews, client interviews, meeting minutes   - *Support the NT RHD Steering Committee within NT Department of Health to coordinate RHD care*   *Who:* Project Officers, NT RHD Steering Committee  *What:* frequent liaison between Project Officers and NT RHD Steering Committee  *How:* via attending meetings, phone calls and emails  *Why:* to enable tracking of the project and input from both parties  *Data:* stakeholder interviews, NT RHD Steering Committee meeting minutes  **DELIVERY SYSTEM DESIGN**   - *Allocate/confirm and document responsibility for ARF/RHD care among HC staff to facilitate planned care interactions and follow-up*   *Who:* HC Manager, Project Officers, HC RHD Coordinator  *What:* Health centre to allocate responsibility to coordinate RHD activities; this can be one or more staff. Once these activities are established then none are very time consuming.  *How:* In discussion with local significant person, HC Manager and Project Officer to select staff and allocate responsibility.  *Why:* The advantage of this approach is that the portfolio responsibility for RHD can move to other staff members if needed, for example in the event of staff turnover. This approach will clarify who takes responsibility for which aspects of RHD care, and how this will be monitored within the HC. It also facilitates effective communication and working relationships with the community.  *Data:* staff interview   \| Activities to be undertaken within the Health centre RHD portfolio  • Define list of ARF/RHD clients each month in consultation with RHD Register  • Track client whereabouts and communicate about clients who have gone elsewhere  • Meet regularly with the HC staff to advise of client care  • Devise a system with reception staff for rapid triage and care of ARF/RHD clients  • Identify each week which clients are due for BPG. Coordinate with staff, including General Practitioner  • Coordinate a regular community RHD meeting (x 3 per year – see below)  • Prioritise and develop ARF/RHD client list to be seen by the visiting specialists and dentist  • Liaise regularly with the NT RHD Control Program staff to foster effective relationships  • Ensure Care Plans for all ARF/RHD clients and troubleshoot any issues arising  • Update Care Plans for all ARF/RHD clients every 18 months  • Keep record of local staff completion of on-line RHD training modules \| \| --- \|  - *Streamline care for ARF/RHD clients*   *ho:* HC RHD Coordinator in collaboration with the Project Officers  *What:* Streamline Care  *How:*   - Devise a site-specific way to ensure all known ARF/RHD clients within the community have this diagnosis flagged in their file (whether paper or electronic). - Make sure that within this file there is a way to record when this person received their injection. - Talk with staff about ‘days at risk’ for injections, so clients can receive injections opportunistically. - Develop a fast tracking system to make sure that for those who come to the health centre just for their injection get it as soon as practicable - Prioritise case management for ARF/RHD clients with poor adherence - Record these activities   *Why:* to increase delivery of efficient and proactive care for RHD clients  *Data:* Routine SP data, staff interview, client interview  **DECISION SUPPORT**   - *Integrate evidence-based guidelines and decision support aids for ARF/RHD into daily clinical practice; ensure HC staff are trained regularly on ARF/RHD care with an emphasis on SP planning and delivery*   *Who:* Project Officer, HC RHD Coordinator, HC Manager, NT RHD Control Program  *What:*   - Completion of online learning package on RHD Australia website (http://www.rhdaustralia.org.au/) is facilitated for all relevant staff, or alternative if internet access not feasible. - Clinical staff familiar with pain minimisation techniques for benzathine penicillin G (BPG) injections, as outlined in the national RHD guidelines. - Australian Guidelines for ARF and RHD management available in Health centre working area for quick reference. - All staff aware that questions arising around any issues to do with ARF/RHD management should be referred to and actioned by HC RHD Coordinator - HC RHD coordinator ensures local knowledge of SP recall procedures and client care plans amongst HC staff   *How:*   - Compilation of staff training record for online learning package - RHD discussion in regular staff meetings - Check HC has copy of Australian Guidelines in Health centre working area.   *Why:* to increase delivery of evidence-based care for RHD clients  *Data:* Routine SP data, staff interview, client interview, list of staff completion or progress with online learning package, presence of Australian Guidelines in Health centre working area.  **CLINICAL INFORMATION SYSTEMS**   - *Monitor performance of practice team and care system in relation to ARF/RHD care using Continuous Quality Improvement (CQI) processes*   *Who:* CQI staff, Project Officer, HC manager, HC RHD Coordinator, NT RHD Control Program  *What:* Implementation of RHD CQI tools  *How:* Follow guidance of CQI staff and procedures  *Why:* to improve local team performance strategies  *Data:* Routine SP data, staff interview, client interview, CQI self-evaluation (clinical audits and systems assessment tools)   - *Establish/refine systems to monitor and report ARF/RHD client data regularly to HC staff to facilitate care planning*   *Who:* Project Manager/Project Coordinator and NT RHD Control Program Coordinator  *What:* To commission IT consultant to work with existing information systems to enable information to be automatically transferred to the jurisdictional register, and for reports to be produced.  *Why:* To provide greater control of the HC over its own data, by enabling the HC staff to receive regular reports of their own performance against the national agreed indicators.  *Data:* staff interview   - *Review and strengthen active systems of reminders and recalls for SP for HC staff and ARF/RHD clients*   *Who:* HC Manager, HC RHD Coordinator, Project Officers  *What:* Develop site specific method to recall clients for their injections.  *How:* Some examples:   - using new moon or full moon as a reminder for next scheduled needle, text messaging, ensuring up-to-date contact numbers for the client and their family members are on file - appointment cards - transporting clients to the HC - identifying different days or weeks for particular family groups to attend the health centre - delivery of BPG injections outside of the health centre, including at home or in schools   *Why:* In order to enhance delivery of SP according to client preferences and health centre systems. There will be variation in how this is done between services, but as a minimum the HC will be expected to develop a system of active recall for SP injections.  *Data:* Routine SP data, staff interview, client interview, description of local recall system  **SELF-MANAGEMENT SUPPORT**   - *Up-skill HC staff in self-management support techniques.*   *Who:* HC staff, NT DoH Health Development group, HC RHD coordinator, research team  *What:* Certain HC staff complete self-management support training  *How*:   - Select staff members to do self-management support training - Use of the online training course for HC personnel being trialed by NT DoH - Project Officers use ‘train the trainer’ guide on patient education with local staff   *Why*: currently self-management support is a less developed component in the care for ARF/RHD clients. This is partially due to limited self-management support experience on the part of the HC staff. Training should increase staff capacity and willingness to boost self-management support initiatives.  *Data:* Routine SP data; staff interview, record of self-management support training.   - *Establish/strengthen group or individual self-management support program for ARF/RHD clients facilitated by HC staff where expertise available*   *Who:* NT DoH Cardiac Coordinators, NT DoH specialist outreach teams, local staff trained in self-management support, HC Manager, HC RHD coordinator, Project Officer, community members  *What:*   - Conduct local self-management support sessions - Enhance client-level understanding of ARF/RHD through facilitated self-management support initiatives and health literacy activities - Explore and enhance the role of local “champions” (prominent people in the community, with or without ARF/RHD, to engage other ARF/RHD clients), and other locally significant groups.   *How:*   - Arrange client/community self-management support sessions using locally agreed upon processes - Arrange client/community RHD education sessions using locally agreed upon processes - Determine what education resources have been used with clients and which, in their opinion, have been effective and what their ongoing perceived learning needs are - A small number of case studies, based on the clinician investigators’ knowledge of individual ARF/RHD case histories, will also be used to inform appropriate approaches to client self-management support   *Why:* to empower RHD clients to better manage their illness and adhere to SP and to increase community support for clients and /or their parents/guardians  *Data:* Client interview, staff interview, stakeholder interviews, record and description of education sessions with clients and/or community and list of local potential community support groups.   - *Investigate sustainable incentives to ARF/RHD clients for adhering to SP*   *Who*: HC RHD Coordinator, HC Manager  *What*: Sustainable incentives that do not require substantial new resources  *How*: Site can develop own ideas – may include:   - Certificates - Involvement in Heart Beads program run by HeartKids Australia. HeartKids Australia has given their approval to participate in this program where health centres are provided bead kits and can give individual beads to children each time they attend the health centre to receive their scheduled injection. - Creation of local age-appropriate support club   *Why*: to empower RHD clients to better manage their chronic illness and adhere to SP  *Data*: Staff interview, client interview, routine SP data, record and description of community RHD support activities  **COMMUNITY LINKAGES**   - *Partner with community organisations to support timely delivery of secondary prophylaxis (SP) to ARF/RHD clients*   *Who:* HC manager, HC RHD coordinator, Project Officers, relevant community organisations  *What:* Community organisations collaborate with HC staff to optimise health service delivery for ARF/RHD clients.   - Project Officers and HC RHD coordinator determine what community groups exist in the local community and their relationship with young people (for example, ranger programs, sport and recreation programs, school programs, youth programs) - Project Officers and HC RHD coordinator determine clan leaders within community - Meet with community groups and clan leaders to determine possible linkages - Case studies (see Self-Management Support) will also inform development of appropriate community linkages.   *Why*: To increase community awareness of and support for ARF/RHD and the importance of adhering to SP  *Data:* Stakeholder interviews, staff interviews, community group description and responses   - *Strengthen health promotion activities in communities*   *Who:* HC RHD Coordinator, HC Manager, Project Officer, NT RHD Control Program, community representatives  *What:* Campaigns targeted at ARF/RHD clients and their parents/guardians.  *How:* Resources provided by RHDAustralia. It is envisaged that the local program will be developed and implemented by a subgroup formed out of the local community RHD working group.  *Data:* Routine SP data, staff interview, client interview, records of health promotion activities |
| --- | --- |
